# Simulation of the ground states of spin rings with cavity-assisted neutral atoms

**DOI:** 10.1038/srep07623

**Published:** 2015-01-05

**Authors:** Peng Xue, Xiang Zhan, Zhihao Bian

**Affiliations:** 1 Department of Physics, Southeast University, Nanjing, Jiangsu 211189, China; 2 State Key Laboratory of Precision Spectroscopy, East China Normal University, Shanghai 200062, China

## Abstract

Quantum phase transitions occur when the ground state of a Hamiltonian undergoes qualitative changes with a control parameter changing. In this paper we consider a particular system—an Isng-type spin ring with competing many-body interactions. Depending on the relative strength interactions, the ground state of the system is either a product state or entangled state. We implement the system in a cavity-assisted neutral atomic simulator and study the non-locality and entanglement of the simulated ground state of an Ising-type three-spin ring with the control parameter changing. The simplicity of the setup and its robustness to noise give it a great practicality within the framework of current experimental technology.

A quantum simulator^1^,^2^,^3^,^4^ is a platform that allows us to reproduce the behaviour of different complex system. The original idea of quantum simulation was proposed by Feynman^5^. The reliable simulation of structural changes due to the ground state of the system would allow to witness the emergence of critical manifestations typical of quantum many-body systems^6^,^7^,^8^,^9^,^10^,^11^,^12^,^13^,^14^,^15^,^16^.

The quantum simulation of spin models can shed light on a variety of open problems such as quantum phase transition^17^, correlated one-dimensional system^18^ and high-T_c_ superconductivity^19^. The entanglement of ground states of spin models is one of the aspects that distinguishes quantum phase transitions from their classical analogs. The study of quantum phase transitions shows interesting connections between two important fields—condensed-matter physics and quantum information science. Depending on the control parameters, the ground state of the many-body spin system is either a product state or entangled state^20^,^21^. In the parameter space these different ground states form a phase diagram with different entanglement phases.

In this paper, we encode the wave function of the ground state of a Ising type multi-spin ring by using cavity-assisted neutral atoms. The effect of a simulated magnetic field related to the control parameter, leading to a critical modification of the relation within the spin ring, is analyzed by studying multi-spin entanglement.

## Results

### Idea

We initially prepare the state of the system in the ground state of the Hamiltonian of an Ising-type spin ring and drive the system from one phase into another different phase. By quantifying different types of entanglement, we can observe the quantum transition induced by the many-body interactions. Our goal is to characterize the fundamental symmetry changes occurring in the ground state of the Ising-type spin ring when the control parameter changes by assessing multipartite nonlocality and entanglement^22^. In order to achieve this aim we calculate the amount of both bipartite and tripartite entanglement in the simulated ground state for various control parameters.

We consider the ground state of an Ising ring governed by the Hamiltonian 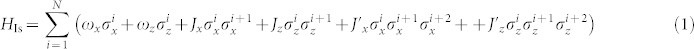
with 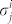 the *j*th Pauli operator of spin *i* and 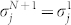, *j* = *x*, *y*, *z*. In Eq. (1), *ω_x_* and *ω_z_* are the magnetic energies of the spin subjected to a global longitudinal and transverse magnetic fields respectively, and *J_x_*, *J_z_*, 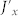, 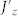 are the two- and three-body inter-spin coupling strengths. For different magnetic energies and coupling strengths, the ground state of the Ising-type spin ring governed by Eq. (1) shows different non-locality and entanglement properties. We consider the two cases with different magnetic energies and coupling strengths *ω_x_*, *ω_z_*, *J_x_*, *J_z_*, 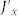, 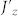:

- Two-body Ising model, i.e., 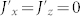. For simplification and not losing the generality, we suppose *J_z_* = *ω_x_* = 0 and then the Hamiltonian becomes 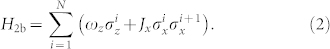
We are able to simulate the ground state of an *N* = 3 Ising ring, which reads 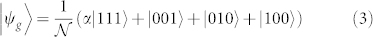
with 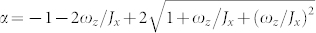 and 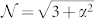. In particular for *ω_z_*/*J_x_* → 0, the ground state of the Ising-type spin ring approaches the Greenberger-Horne-Zeilinger (GHZ) state^23^,^24^,^25^,^26^ with the coefficient *α* → 1. For *ω_z_*/*J_x_* → ∞, the ground state tends to be a W state^27^,^28^,^29^,^30^,^31^ with *α* → 0. Whereas, for *ω_z_*/*J_x_* < 0, the coefficient *α* is almost linear dependent on *ω_z_*/*J_x_* and for |*ω_z_*/*J_x_*| → ∞ the ground state tends to be a product state.
- Three-body Ising model, i.e., *J_x_* = *J_z_* = 0. For simplification and not losing the generality, we suppose 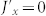 and then the Hamiltonian becomes 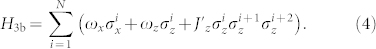
Based on the zero-order perturbation theory, the ground state for *N* = 3 can be calculated as 
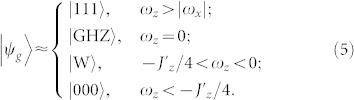
We now show how to simulate the ground state of the Ising-type three-spin ring in Eq. (3) and investigate the fundamental symmetry changes occurring in the ground state by assessing multipartite non-locality and entanglement. By local operation and classical communication (LOCC), the three-qubit GHZ state can be rewritten as |GHZ〉 = (|111〉 + |001〉 + |010〉 + |100〉)/2. It is obvious that the GHZ state is a superposition of the W state and a product state |111〉.

A positive operator-valued measure (POVM) defined as 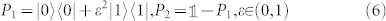
is then applied to each of the particles which are prepared firstly in the GHZ state. If for all particles we obtain the outcome *P*_1_, the resulting state simulates the ground state of the Ising-type three-spin ring with *α* = *ε*^2^

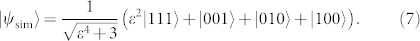
The probability of getting the (desired) outcome 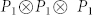 is (*ε*^6^ + 3*ε*^2^)/4 and the corresponding similarity to the GHZ state, W state and product state are respectively 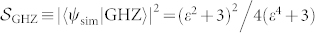, 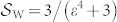, and 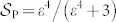. For *ε* → (0, 1), 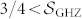, 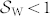, and 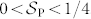. So, for *ε* → 1 the POVM does nothing and the GHZ state does not change. Whereas, as expected, if *ε* tends to 0, 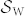 goes to 1 while the probability of success goes to 0 at the same time, i.e. the probability that one can get the outcome *P*_1_ on each particle goes to 0. For the control parameter *ε* ∈ (0, 1), the maximal similarity to the product state 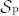 is only 1/4 and thus the simulated ground state shows non-locality.

Our goal is to characterize the fundamental properties of the ground state of the Ising ring when the control parameter such as *ε* changes by assessing multipartite non-locality and entanglement. We perform our characterization of the quantum correlation of the model starting for an assessment of the bipartite entanglement between any two spins by tracing out one and obtain the reduced density operators *ρ*_12_, *ρ*_23_ and *ρ*_13_ (here *ρ*_23_ = Tr_1_ (|*ψ*_sim_〉 〈*ψ*_sim_|)). From the density operator we can calculate the concurrence 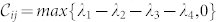 with *λ_k_* the square root of the eigenvalues of 
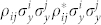
 as a bipartite entanglement measure and obtain 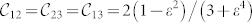, which is non-zero with *ε* ∈ (0, 1). Thus, we show for the simulated ground state of the Ising ring there exists a non-zero amount of bipartite entanglement and the amount changes with the control parameter *ε*. With *ε* increasing from 0 to 1 the concurrence decreases from 2/3 to 0.

Then we focus on the critical structural changes on the multipartite non-locality of the simulated ground state |*ψ*_sim_〉. This is done by detecting the 3-qubit entanglement with linear witness requiring measurement of fewer elements of the Pauli operator. For example, the 3-tangle 
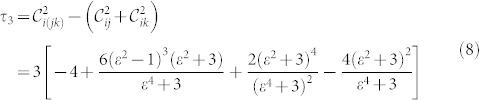
with the bipartite concurrence 
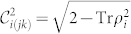
 measures the entanglement of a pure three-qubit state. It is also possible to estimate the value of measures of genuine tripartite entanglement. For the simulated ground state of the Ising model we notice that *τ*_3_ provides a complete refutation of local realism within the regime *ε*. However the value of *τ*_3_ is strongly determined by the similarity between the simulated ground state and a GHZ state. Whereas it is not valid if the simulated ground state is a W state. Thus we choose another choice of the measure of genuine tripartite entanglement—the violation of the Svetlichny inequality^21^,^32^. For the simulated ground state of a three-spin ring, it becomes 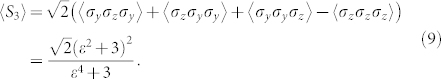
The inequality |〈*S*_3_〉| ≤ 4 is violated for any genuine tripartite entangled state. For the simulated ground state of the spin ring, the inequality is violated for 0 ≤ *ε* < 1.834.

### Implementation

**Figure 1:**
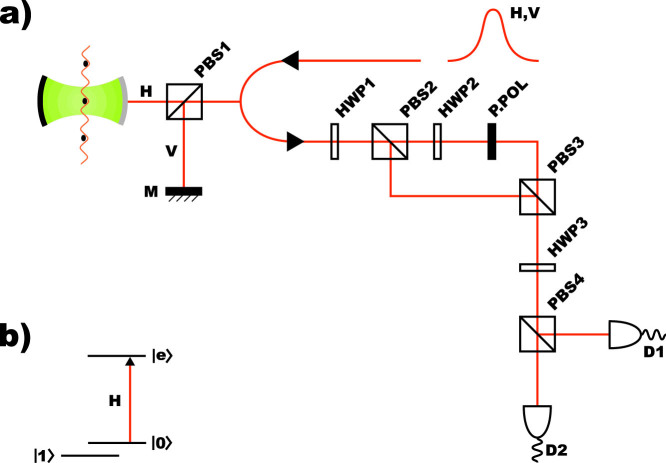
Schematic setup. (a) Simulation of the ground state of the Ising-type spin ring with the cavity-assisted neutral atoms via photon-scattering. Single atoms are taken in and out of the cavity by moving the optical lattice. After the reflection by the cavity scattered photon pulses leak out and pass through the linear optical elements including HWPs and PBS. They are finally detected by two single-photon detectors. The partial polarizer (P. POL) implements the certain POVM on atoms through post-selection. For a click in the detector *D*1, the outcome *P*_1_ is obtained directly; a click in *D*2 means that we obtain *P*_1_ after a single-qubit rotation on the atom. (b) Relevant three-level atomic structure and the coupling configuration between the energy levels.

For the physical implementation we choose—in contrast to the use of photons like in refs. 21, 33—neutral atoms with long coherent life time. Neutral atoms are trapped in both a transverse optical lattice and in a cavity at the same time shown in Fig. 1a. This system can allow the neutral atoms to be taken in and out of the cavity in order to avoid the requirement for individual-qubit addressing. A three-level *λ*-type atom is shown in Fig. 1b. Atomic states |0〉 and |1〉 are two stable ground states. The atomic transition from |0〉 to the excited state |*e*〉 is resonantly coupled to a cavity mode *a_c_*. The state |1〉 is decoupled to the cavity due to a large hyperfine splitting.

The details of our method for generating three-atom GHZ states via cavity-assisted interaction can be found in our recent work^34^. Each atom is prepared in the superposition state 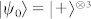, where 
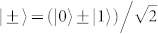
, and we apply a controlled phase flip operation on each pair of them and hence get |*ψ*_t_〉 = *U*(*t*) |*ψ*_0_〉 = [|+〉 (|00〉 + |11〉) + |−〉 (|01〉 + |10〉)]/2, where the unitary evolution operator *U*(*t*) can equivalently be described by a product 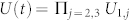 of commuting controlled-z (CZ) gates *U*_1,*j*_ ≡ diag (1, 1, 1, −1)_1,*j*_ acting on pairs of qubits. By applying the single-qubit rotation (|1〉_1_ 〈+| + |0〉_1_ 〈−|) on |*ψ*_t_〉, we obtain a GHZ state.

The aforementioned POVM can be implemented by successfully reflecting a single photon pulse from the optical cavity and the subsequent usage of linear optical elements. Initially, the atom is prepared in the superposition state *α* |0〉 + *β* |1〉 with |*α*|^2^ + |*β*|^2^ = 1, and the single-photon pulses are in the state 
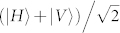
 with |*H*〉 (|*V*〉) the horizontal (vertical) polarization state of single photons. Assume that the cavity mode is horizontal and resonantly driven by the horizontal polarized photons. The vertical polarized photons are then reflected by the mirror *M*.

We now present a theoretical model to demonstrate that the POVM can be implemented on atoms through photon scattering. In the rotating wave approximation, the Hamiltonian of atom-cavity and free space in the rotating frame is (setting 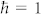) 
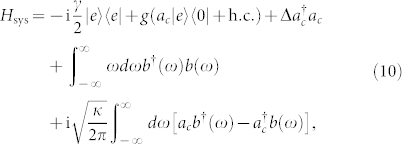
where *γ* is the rate of spontaneous decay of the excited state |*e*〉, Δ denotes the detuning between the cavity field mode *a_c_* and the atomic transition, and *b*(*ω*) with the standard relation [*b*(*ω*), *b*^†^(*ω*′)] = *δ* (*ω* − *ω*′) denotes the one-dimensional free-space modes which couple to the cavity mode *a_c_*. According to the quantum Langevin equation and the boundary condition of the cavity, we can deduce that the single-sided cavity input and output field operators *b*_in_ (*t*) and *b*_out_(*t*) are connected with the cavity mode *a_c_*(*t*) through the relations^35^,^36^,^37^,^38^,^39^,^40^,^41^,^42^,^43^

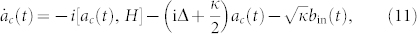
where 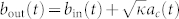, and the Hamiltonian 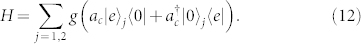
describes the coherent interaction between the atom and cavity mode *a_c_*. The time-dependent operators *b*_in_(*t*) and *b*_out_(*t*) satisfy the commutation relations 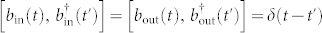. For the atom in |1〉, the Hamiltonian *H* does not work. Thus based on Eqs. (10) and (11) we obtain 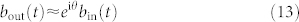
with 
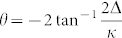
, if the input pulse shape changes slowly with time *t* compared with the cavity decay rate *κ*. In the case of resonant interaction Δ = 0, we have *b*_out_(*t*) ≈ −*b*_in_(*t*). That means if the state of the atom is in |1〉, the output optical field acquires the phase *π* after the interaction. In another case |0〉, the effective detuning between two dressed cavity modes and the input single-photon pulse are Δ = ±*g*. If 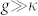, the phase of the output pulse is *θ* ≈ 0. From the description above, we conclude that the state of the whole system of atom-cavity and free-space acquires the phase *π* or 0, after the pulse is reflected by the cavity. This evolution can be characterized by (*α* |0〉 + *β* |1〉)|*H*〉 → (*α*|0〉 − *β*|1〉)|*H*〉, where we have discarded the state of cavity since it is always in the vacuum state, and |*V*〉 denotes the state of free-space photon.

The net effect of these two subprocesses is that the reflection of a single-photon pulse from the cavity actually performs a control operation exp (i*π* |1*H*〉 〈1*H*|) on the atom and the single photon.

According to Fig. 1, after leaving the cavity, the photons are injected polarizing beam splitter 1 (PBS1), which reflects vertically polarized photons and transmits horizontally polarized ones. The vertical photons are then reflected by a mirror and PBS1 and then injected into the optical mode with the horizontally photons which are transmitted by PBS1. A Hadamard operation is applied on the polarizations of the photons by the half wave plate 1 (HWP1) with the angle between the optical axis and horizontal setting to *π*/8, which performs 
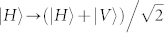
 and 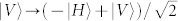. Then we obtain the state *α*|0〉|*H*〉 + *β*|1〉|*V*〉. The photons are injected into PBS2, subsequently following by *π*/8-oriented HWP2 which applies a Hadamard operation on the polarization of the photon reflected by PBS2. Thus the applied partial polarizer operates in the basis {|*H*〉, |*V*〉}. Then the photons reflected by PBS3 passe HWP3 and PBS4 and are then detected by single-photon detectors. The resulting state is *α*|0〉 + *βε*|1〉 (not normalized). Beginning with the ideal GHZ state, we simulate the ground state of the Ising-type spin ring, given that the three POVMs yield *P*_1_.

### Feasibility

We do not require the particularly assumptions which have been made for the experimental parameters. The relevant cavity QED parameters used for simulation are assumed to be (*g*, *κ*, *γ*)/2*π* = (27, 2.4, 2.6) MHz and satisfy the condition for strong coupling regime 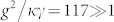. The cavity consists of two 1-mm-diam mirrors with 10 cm radii of curvature separated by 75 *μ*m^44^. Suppose that the wavelength of the cavity mode is ~780 nm. The distance *d* between two atoms in an optical lattice is ~10 *μ*m, which is larger than the waist of the cavity mode ~ 5 *μ*m. Thus only one atom stays inside the cavity for the logical gate operations and the others outside are not affected. The evolution of two atomic states is accomplished during the time which takes the single-photon pulse to pass through the cavity *T* ~ 200/*κ* = 10 *μ*s. The maximum velocity of the atoms in the optical lattice is about 30 cm/s and the maximum acceleration imparted is 1.5 g. It takes a time *τ*_T_ ≈ 100 *μ*s to move the proper atoms into and out of the cavity. The major decoherence here is dephase. The coherence time of atoms lasts 1–100 ms, which depends on the sensitivity to the magnet fluctuations of the internal atomic states^45^,^46^,^47^,^48^. Thus both the gate preformation and the transport of atoms can be done within the coherent time.

The transmission probability *T*_H_ = 0.89 and *T*_V_ = 0.18 respectively are obtained by applying the partial polarizer with proper settings^49^. Thus we have 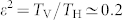.

The major sources of noise and decoherence in the scheme of quantum information processes via cavity QED system are usually found to be: addressing errors, spontaneous emissions of the atoms and long term interferometric phase instability. Our system can allow the atoms to be taken in and out of the cavity so that no individual-qubit addressing is required. Remarkably, in our scheme only local operations are used and hence there is no interferometer required. That means our method does not suffer from the last mentioned problem. On the other hand, spontaneous emissions of the atoms only lead to photon losses and merely decrease the probability of success. However no contribution due to atomic spontaneous emission to a lack of fidelity.

**Figure 2:**
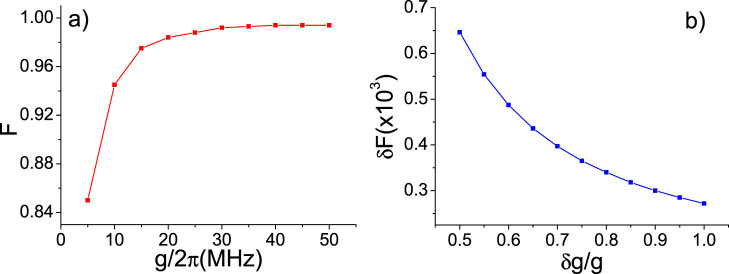
Numerical simulations of the fidelity and the change of the fidelity. (a) The fidelity of the simulated ground state versus *g*/2*π* with pulse duration *T* = 10 *μ*s, *κ*/2*π* = 2.4 MHz, *γ*/2*π* = 2.6 MHz, *ε*^2^ = 0.2. For the numerical simulation a Gaussian shape for the input pulse with *f*(*t*) ∝ exp [−(*t* − *T*/2)^2^/(*T*/5)^2^] have been used. (b) The change of the fidelity 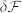 as a function of the change of the coupling *δg*/*g*.

There are also some minor contributions to the fidelity degradation which have been estimated by numerical simulations. For example the shape mismatching between the input and output pulses can also cause the lack of fidelity. Figure 2a shows that a high fidelity is obtained with the parameters (*κ*, *γ*)/2*π* = (2.4, 2.6) MHz and *ε*^2^ = 0.2—which means that 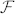 is up to 99% for *g*/2*π* > 27 MHz^44^. The randomness in the coupling rates caused by fluctuations in the position of the atom also decreases the fidelity of our scheme, which can be determined by the variation of *g*. In Fig. 2b the fidelity 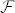 is shown to be insensitive to the randomness in *g*, as 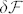 which describes the fluctuation of the fidelity, stays below 10^−3^ for *g* varying from 27 MHz to 13.5 MHz.

## Conclusion

We have presented a proposal to simulate the ground state of an Ising-type three-spin ring. Ising spin rings, which have been investigated in detailed in solid-state physics, play an important role in quantum information processing. Depending on the control parameter, the ground state of the Ising-type three-spin ring is either of a product state, GHZ state, or W state. We characterize the fundamental symmetry changes occurring in the ground state of the Ising-type spin ring when the control parameter changes by assessing multipartite non-locality and entanglement.

Using the cavity-assisted neutral atoms as quantum simulator, we have confirmed that in this system the simulated ground state undergoes a quantum transition of different multipartite entangled state with the control parameter changing. The system limits the control parameter in the regime *ε* ∈ (0, 1), for the simulated ground state the maximal similarity to the product state is small enough and thus shows non-locality. Hence we study multipartite non-locality and entanglement in the simulated ground state of an Ising ring. Remarkably, our proposal releases the requirement of an interferometer. Therefore our method does not suffer from the problem of long term interferometric phase stability, and the impact of changes in the path lengths is reduced considerably. The robustness of this scheme has been shown through exact simulations with experimental parameters that incorporate various sources of noise found in experiments such as photon losses, shape mismatching between the optical pulses at input and output as well as the fluctuations in the position of the atom. The simplicity of the setup and its robustness give it a great practicality within the framework of current experimental technology.

## Methods

### Realization of collective CZ gate on two atoms

To perform a collective CZ gate on two atoms, firstly a single-photon pulse in its state |*p*〉 is reflected from the cavity, which is resonant with the bare cavity mode. For a sufficient long pulse, the reflection of that pulse from a resonant cavity keeps the shape of pulse almost unchanged but flips a global phase of the pulse. Hence this operation is performed in the limit with 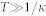 (here *T* is the pulse duration and *κ* is the cavity decay rate). There are two cases. Firstly if both atoms are in the state |1〉, atom-cavity coupling is negligible and no shift of the frequency of the bare cavity mode. After resonant reflection, the atom-photon state |1〉_1_|1〉_2_|*p*〉 obtains a global phase and evolves into −|1〉_1_|1〉_2_|*p*〉. For the second case in which either or both of the atoms are in the state |0〉, the effective frequency of the dressed cavity mode is then shifted due to the atom-cavity coupling, which is described by the Hamiltonian *H* shown in Eq. (12). If the coupling rate satisfies 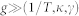 with *γ* the rate of spontaneous decay of the excited state |*e*〉, then the frequency shift is as large as *g*. The incident single-photon pulse is then reflected by an off-resonant cavity. Hence, both shape and phase keep unchanged for the reflected pulse. Thus, the atom-photon states |0〉_1_ |0〉_2_ |*p*〉, |0〉_1_ |1〉_2_ |*p*〉, and |1〉_1_ |0〉_2_ |*p*〉 remain unchanged.

### Realization of partial polarizer

The partial polarizer can be realized by inserting into one path a series of coated glass, tilted about the vertical axis by 58° (approximately Brewster's angle for these slabs). After the slabs, the transmitted photons are vertically polarized. Ideally, the partial polarizer transmits vertical photons perfectly and reflects the horizontal photons partially. The transmission rate for horizontal photons is *T*_H_ = 1, while for vertical photons only *T*_V_ = *ε*^2^.
